# Enhanced Nerve Regeneration by Exosomes Secreted by Adipose-Derived Stem Cells with or without FK506 Stimulation

**DOI:** 10.3390/ijms22168545

**Published:** 2021-08-09

**Authors:** Cheng-Shyuan Rau, Pao-Jen Kuo, Shao-Chun Wu, Lien-Hung Huang, Tsu-Hsiang Lu, Yi-Chan Wu, Chia-Jung Wu, Chia-Wei Lin, Chia-Wen Tsai, Ching-Hua Hsieh

**Affiliations:** 1 Department of Neurosurgery, Kaohsiung Chang Gung Memorial Hospital and Chang Gung University College of Medicine, Kaohsiung 83301, Taiwan; ersh2127@adm.cgmh.org.tw (C.-S.R.); ahonbob@gmail.com (L.-H.H.); 2 Department of Plastic and Reconstructive Surgery, Kaohsiung Chang Gung Memorial Hospital and Chang Gung University College of Medicine, Kaohsiung 83301, Taiwan; bow110470@gmail.com (P.-J.K.); rabbit670326@yahoo.com.tw (T.-H.L.); janewu0922@gmail.com (Y.-C.W.); alice8818@yahoo.com.tw (C.-J.W.); sallylin1201@gmail.com (C.-W.L.); flying011401@gmail.com (C.-W.T.); 3 Department of Anesthesiology, Kaohsiung Chang Gung Memorial Hospital and Chang Gung University College of Medicine, Kaohsiung 83301, Taiwan; shaochunwu@gmail.com; 4 Center for Vascularized Composite Allotransplantation, Chang Gung Memorial Hospital, Taoyuan 33305, Taiwan

**Keywords:** sciatic nerve crush injury, peripheral nerve regeneration, exosome, adipose-derived stem cells (ADSC), tacrolimus (FK506), proteomic analysis

## Abstract

Exosomes secreted by adipose-derived stem cells (ADSC-exo) reportedly improve nerve regeneration after peripheral nerve injury. Herein, we investigated whether pretreatment of ADSCs with FK506, an immunosuppressive drug that enhances nerve regeneration, could secret exosomes (ADSC-F-exo) that further augment nerve regeneration. Designed exosomes were topically applied to injured nerve in a mouse model of sciatic nerve crush injury to assess the nerve regeneration efficacy. Outcomes were determined by histomorphometric analysis of semi-thin nerve sections stained with toluidine blue, mouse neurogenesis PCR array, and neurotrophin expression in distal nerve segments. Isobaric tags for relative and absolute quantitation (iTRAQ) were used to profile potential exosomal proteins facilitating nerve regeneration. We observed that locally applied ADSC-exo and ADSC-F-exo significantly enhanced nerve regeneration after nerve crush injury. Pretreatment of ADSCs with FK506 failed to produce exosomes possessing more potent molecules for enhanced nerve regeneration. Proteomic analysis revealed that of 192 exosomal proteins detected in both ADSC-exo and ADSC-F-exo, histone deacetylases (HDACs), amyloid-beta A4 protein (APP), and integrin beta-1 (ITGB1) might be involved in enhancing nerve regeneration.

## 1. Introduction

Despite advances in microsurgical techniques and agents that enhance nerve regeneration, treatment outcomes for peripheral nerve injury remain unsatisfactory [1,2]. For example, the immunosuppressive drug FK506 has been shown to possess neuroprotective and neurotrophic actions that can accelerate nerve regeneration [3,4,5], as well as enhance nerve regeneration following nerve allotransplantation and nerve crush injury in clinical settings [6,7,8,9]. However, the precise mechanism mediating the neuroregenerative effect of FK506 remains unclear, and its potential side effects, including nephrotoxicity, hyperglycemia, and central nervous system toxicity, restrict its widespread use in peripheral nerve injury [3].

Currently, cell-based therapy is considered promising for treating peripheral nerve injury [10,11,12]. Adipose-derived stem cells (ADSCs), known to originate from stromal-vascular fragments of adipose tissue, reportedly possess promising therapeutic potential [13]. Several studies have demonstrated that ADSCs can promote peripheral nerve regeneration [12,14,15,16]. Furthermore, conditioned medium from ADSCs was found to enhance axonal regeneration [17,18]. Secretomes in the medium reportedly contain secreted proteins, as well as exosomes, small extracellular vesicles that can mediate intercellular communication by transporting proteins, nucleic acids, and lipids into target cells, thus altering the behavior of recipient cells [19,20]. Exosomes secreted by ADSCs (ADSC-exo) might enhance nerve regeneration by increasing remyelination [21], stimulating Schwann cell proliferation [22], and suppressing neuronal autophagy and apoptosis [23]. In addition, neural growth factors, including nerve growth factor (NGF), brain-derived neurotrophic factor (BDNF), insulin-like growth factor-1 (IGF-1), fibroblast growth factor-1 (FGF-1), and glial cell-derived neurotrophic factor (GDNF), have been detected in ADSC-exo, partially clarifying their ability as a therapeutic tool for nerve regeneration [22].

In the present study, we aimed to determine whether exosomes secreted by ADSCs following FK506 stimulation (ADSC-F-exo) could further enhance nerve regeneration when compared with ADSC-exo. Accordingly, we employed a mouse model of sciatic nerve crush injury to assess the efficacy and outcomes of prepared exosomes on nerve regeneration. Furthermore, we employed isobaric tags for relative and absolute quantitation (iTRAQ) of the protein content of designed exosomes to determine the potential exosomal proteins mediating nerve regeneration following peripheral nerve injury.

## 2. Results

### 2.1. Characterization of Isolated Exosomes

**Figure 1:**
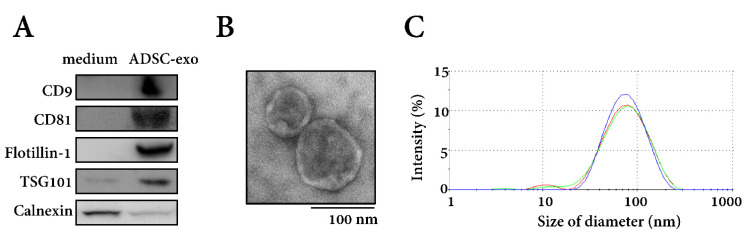
Characterization of isolated exosomes by (**A**) Western blotting for exosomal surface markers, (**B**) transmission electron microscope analyses, and (**C**) the measurement of particle diameter by dynamic light scattering. ADSC-exo, exosomes secreted by adipose-derived stem cells (ADSCs).

According to western blotting results, isolated ADSC-exo expressed positive exosomal surface markers, including CD9, CD81, flotillin-1, and TSG101, with no expression of negative control protein calnexin, when compared with proteins isolated from the culture medium (Figure 1A). Transmission electron microscopy (TEM) revealed that the exosomes were composed of lipid bilayers, displaying a cup-shaped appearance with acceptable quality in terms of morphology and size range (Figure 1B). Based on dynamic light scattering (DLS) measurements, the exosomes’ size distribution showed a single peak, with an average size of 79.8 ± 36.9 nm and a polydispersity index (PDI) of approximately 0.74 (Figure 1C). The quality of the isolated exosomes was good with a relatively uniform size distribution.

### 2.2. ADSC-exo and ADSC-F-exo Enhanced Nerve Regeneration

**Figure 2:**
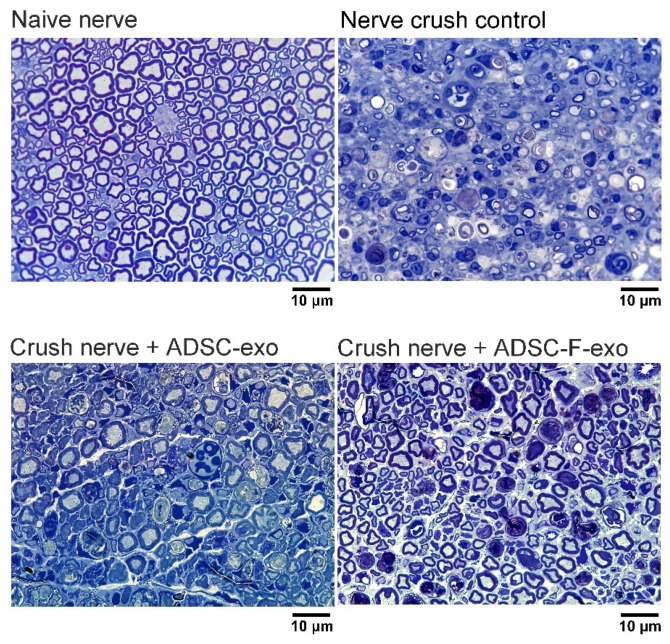
Histomorphology of toluidine blue-stained nerve specimens, harvested 5-mm distal to the injured site in C57BL/6 mice on post-crush day 10. Representative histological sections (×1000) stained with toluidine blue. The magnification bars represent 10 μm. ADSC-exo, exosomes secreted by adipose-derived stem cells (ADSCs). ADSC-F-exo, exosomes secreted by ADSCs following FK506 stimulation.

Figure 2 illustrates toluidine blue-stained semi-thin sections of the axial nerve, harvested 5 mm distal to the injured site, from 6 mice groups on postoperative day 10. According to histomorphometric analysis, treatment of nerve crush injuries with either ADSC-exo or ADSC-F-exo enhanced nerve regeneration when compared with injuries treated with PBS in nerve crush control mice. We recorded a significantly larger fiber width, axon width, fiber area, axon area, myelin area, and total fiber area in nerve crush mice treated with locally sprayed ADSC-exo or ADSC-F-exo than those in nerve crush control mice (Table 1). No significant difference in nerve regeneration was observed between mice treated with ADSC-exo and ADSC-F-exo. However, the nerve regeneration remained suboptimal when compared with the naïve nerve that did not undergo crush injury (Table 1).

### 2.3. Expression of Neurogenesis-Related Genes and Neurotrophins in the Distal Nerve Segment

**Figure 3:**
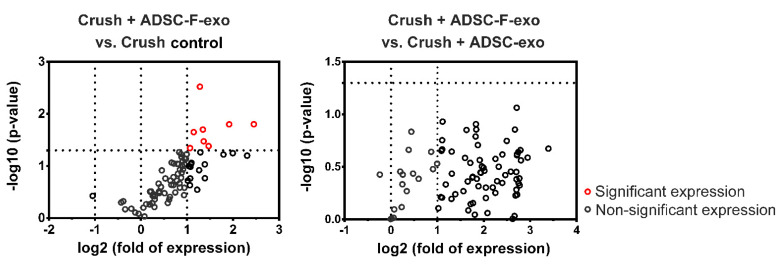
Representative plot of differentially expressed genes in the distal nerve segment of C57BL/6 mice treated with ADSC-F-exo vs. PBS (**left graph**) or ADSC-exo (**right graph**) determined by PCR array on post-crush day 2. Genes showing at least 2-fold differential expression and *p* < 0.05 between experimental and nerve crush control groups are considered significantly upregulated (indicated in red). Statistical analysis was performed by one-way analysis of variance with a post-hoc Fisher’s least significant difference test.

**Figure 4:**
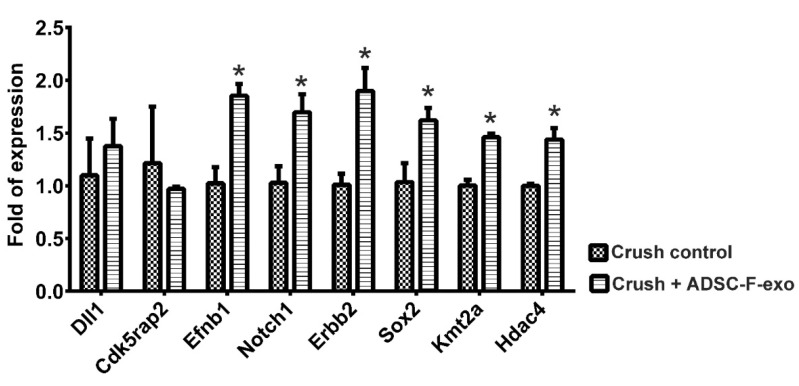
RT-qPCR was performed to validate upregulated genes detected in nerve specimens on post-crush day 2 following ADSC-F-exo treatment vs. those in nerve crush control nerve. Upregulated genes were identified according to the mouse neurogenesis PCR array. RT-PCR, quantitative reverse transcription-PCR analysis. Statistical analysis was performed by one-way analysis of variance with a post-hoc Fisher’s least significant difference test. * indicated a statistical significance which was set at *p* < 0.05.

**Figure 5:**
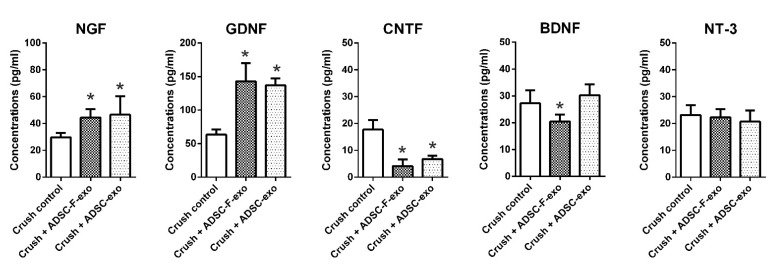
Neurotrophin levels in nerve segments after exosome treatment determined by enzyme-linked immunosorbent assay. Statistical analysis was performed by one-way analysis of variance with a post-hoc Fisher’s least significant difference test. * indicated a statistical significance which was set at *p* < 0.05.

At post-crush day 2, the mouse neurogenesis PCR array revealed that treatment with ADSC-F-exo significantly upregulated eight genes (*Dll1*, *Cdk5rap2*, *Efnb1*, *Notch1*, *Erbb2*, *Sox2*, *Kmt2a*, and *Hdac4*) in the distal nerve segment when compared with nerve crush control mice (Figure 3). The PCR array showed no significant difference between genes expressed in ADSC-exo- and ADSC-F-exo-treated mice (Figure 3). Among these eight genes, six (*Efnb1*, *Notch1*, *Erbb2*, *Sox2*, *Kmt2a*, and *Hdac4*) were significantly upregulated, as validated in the quantitative reverse transcription-PCR analysis (RT-qPCR) (Figure 4). Based on enzyme-linked immunosorbent assay (ELISA) results, treatment with ADSC-exo and ADSC-F-exo significantly increased protein expression levels of NGF and GDNF but decreased expression of ciliary neurotrophic factor (CNTF) in the nerve segment when compared with nerve crush control mice (Figure 5). However, BDNF expression was significantly reduced in ADSC-F-exo-treated mice but not ADSC-exo-treated mice when compared with nerve crush control mice. Neurotrophin-3 (NT-3) expression in nerve segments did not differ significantly among the three mice groups.

### 2.4. Exosomal Protein Content

**Figure 6:**
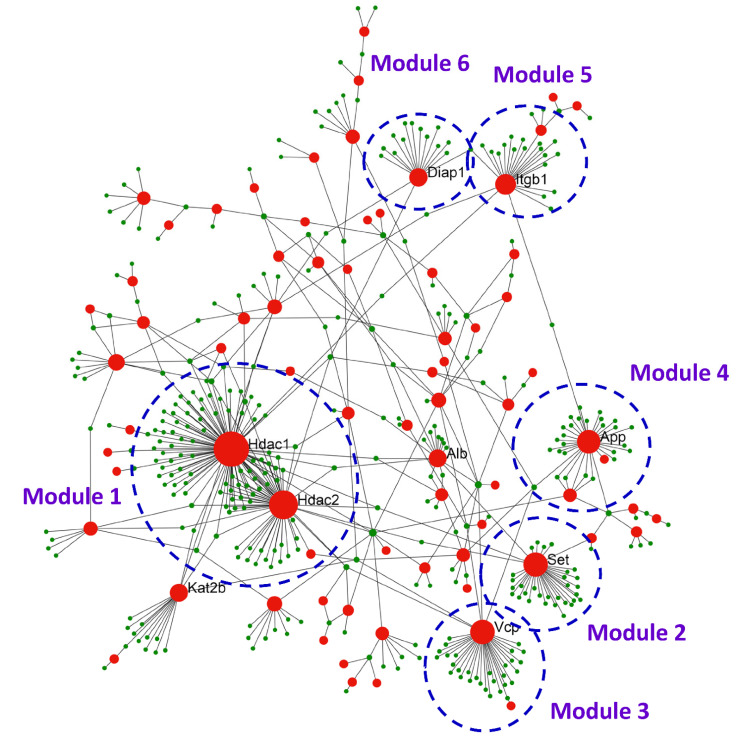
The PPI network of exosomal proteins constructed by Cytoscape presenting the hub proteins, known to possess the highest degree of connectivity among the protein targets, and six key modules network. PPI, protein–protein interaction.

An iTRAQ-based quantitative proteomic analysis was used to analyze expressed exosomal proteins in ADSC-exo and ADSC-F-exo samples (*n* = 2). The exosomes were labeled with 4-plex iTRAQ reagents of varying masses (114–117). In total, 1697 proteins were identified, with 192 exosomal proteins noted at the intersection of all four samples (File S1). The biological functions of the upregulated genes were determined using the Gene Ontology and Kyoto Encyclopedia of Genes and Genomes databases. The upregulated genes were particularly enriched in the following top ten pathways: focal adhesion (17 proteins), human papillomavirus infection (16 proteins), PI3K-Akt signaling pathway (15 proteins), extracellular matrix-receptor interaction (14 proteins), metabolic pathways (13 proteins), pathways in cancer (13 proteins), complement and coagulation cascades (11 proteins), regulation of actin cytoskeleton (10 proteins), lysosome (10 proteins), and rheumatoid arthritis (9 proteins). We imported the protein–protein interaction (PPI) data into Cytoscape and constructed a PPI network of exosomal proteins (Figure 6) to disclose the top nine hub proteins, defined as proteins carrying the highest degree of connectivity. These nine hub proteins included histone deacetylase 1 (HDAC1), histone deacetylase 2 (HDAC2), SET nuclear proto-oncogene (SET), albumin (ALB), amyloid-beta A4 protein (APP), transitional endoplasmic reticulum ATPase (VCP), histone acetyltransferase KAT2B (KAT2B), integrin beta-1 (ITGB1), and protein diaphanous homolog 1 (DIAPH1). Among these, four proteins, including HDAC1, HDAC2 [24,25,26,27,28,29,30,31], APP [32,33,34], and ITGB1 [35,36,37,38,39,40], are known to be involved in nerve regeneration. Using the MCODE plugin with default criteria, six modules, presented in descending order based on MCODE scores, were obtained. These six modules were selected for visualization of the module network (Figure 6). Specifically, HDAC1 and HDAC2 were predicted as key modulators module 1 in the PPI network and consists of 105 genes. SET, VCP, APP, ITGB1, and DIAPH1 are the key modulators in modules 2 to 6, consisting of 30, 30, 26, 20, and 14 genes, respectively.

## 3. Discussion

Herein, our findings revealed that topically sprayed ADSC-exo or ADSC-F-exo at the crush site significantly enhanced nerve regeneration in a mouse model of nerve crush injury. Treatment with either ADSC-exo or ADSC-F-exo increased NGF and GDNF expression in the nerve segment, along with enhanced expression of some neurogenesis-related genes. However, pretreatment of ADSCs with FK506 failed to generate exosomes (ADSC-F-exo) carrying more potent molecules for enhanced nerve regeneration. The proteomic analysis of the intersectional content within ADSC-secreted exosomes, both in the presence and absence of FK506 stimulation, revealed that three exosomal proteins, including HDAC, APP, and ITGB1, may mediate the enhanced nerve regeneration.

HDACs are antagonistic enzymes that regulate gene expression via acetylation and deacetylation of histone proteins, around which DNA is found to be wrapped within the cell nucleus [41]. Members of the HDAC family deacetylate tubulin and actin cytoskeleton components, thus impacting neurite formation [42]. In addition, HDAC1 and HDAC2 reportedly regulate dendrite targeting in the Drosophila olfactory system [43]. Neuronal progenitors lacking HDAC1 and HDAC2 are unable to differentiate into mature neurons and undergo cell death [44]. In rats, HDAC1 is reportedly involved in the axotomy-induced injury of glial cells and dorsal root ganglia [26]. In rat cortical neurons, HDAC2 was shown to regulate dendrite development in response to BDNF, which induces S-nitrosylation of HDAC2 and nitric oxide synthesis, resulting in dendritic growth and branching [45]. Furthermore, it has been reported that the HDAC-mediated deacetylation of nuclear factor-kappa B (NF-κB) is critical for Schwann cell myelination [46]. The loss of both HDAC1 and HDAC2 disrupts neural precursor differentiation, resulting in aberrant brain development [44]. Although HDACs play important roles in neurite growth, HDAC inhibitors attenuated neuronal death and promoted neurite outgrowth and axonal regeneration [29]. Accordingly, it has been suggested that specific HDACs can play distinct roles depending on the developmental stage and effect via histone post-translational modifications [25]. Therefore, the identification of signaling mechanisms mediated by HDAC1 and HDAC2, both detected within ADSC exosomes, is critical for the further understanding and development of treatment approaches targeting nerve crush injury.

APP is a major component of vascular amyloid and plaque present in the brain tissue of patients with Alzheimer’s disease [47]. In familial Alzheimer’s disease, a mutation in APP can result in defective neurite extension [48]. APP is secreted into the medium by most cultured cells and can function as an autocrine factor to induce neurite extension through cell-surface binding [49]. Reportedly, APP overexpression in mice following peripheral nerve injury prevented neuropathic pain [50,51] and motoneuron death [50]. In addition, the β-site APP cleaving enzyme 1 (BACE1), a sole β-secretase generating APP, is crucial for axonal and Schwann cell remyelination of injured nerves [52], and the genetic deletion of BACE1 leads to increased nerve regeneration [53].

Integrins are heterodimeric transmembrane receptors that mediate cell–cell interactions, as well as interactions between cells and the extracellular matrix [54]. During peripheral nerve regeneration, the growth cones of neurons are in contact with basal lamina channels, known to contain laminin [55]. Neuronal responses to laminin are dependent on specific cell-surface receptors, such as integrin. Integrins containing a β1 subunit (ITGB1) bind various collagens and laminins expressed during peripheral nerve regeneration [56]. During the first two weeks following a peripheral nerve injury, endoneurial cells proliferate and express integrin β1 for collagen types I and III. The failure of endoneurial fibroblasts to express the integrin β1 subunit may indicate advanced degeneration of the denervated distal stump [40]. Furthermore, integrin β1 reportedly plays important functional roles in axon outgrowth during development and regeneration [39].

Neurotrophins have long been identified as drivers of neurogenesis during nervous system development and regeneration [57]. Neurotrophic factors, including NGF, GDNF and BDNF, play significant roles in promoting axonal regeneration [58]. As the expression of endogenous neurotrophic factors declines, the regenerative capacities of axotomized neurons and denervated Schwann cells to support regenerating neurons also decrease [58]. Herein, we observed that both ADSC-exo and ADSC-F-exo significantly increased the expression of NGF and GDNF in the nerve segments of crush injury mice when compared with those of nerve crush control mice. BDNF expression was lower in mice treated with ADSC-F-exo than in nerve crush control mice; however, this effect was not observed in mice treated with ADSC-exo. Additional studies are critical for clarifying the impact of these dysregulated neurotrophins on peripheral nerve regeneration. In addition, the detailed mechanism underlying the effect of ADSC-exo treatment on crush nerve injury needs to be elucidated.

Some limitations of this study should be acknowledged. First, 192 exosomal proteins, with 9 hub proteins, were identified based on the proteomic approach and PPI analysis. It cannot be excluded that the neuroregenerative function of some exosomal proteins remains undetected. Furthermore, the effect of exosomes secreted from the ADSCs on the nerve regeneration may rely on factors other than the protein cargo inside exosomes, such as microRNAs [59,60] or long noncoding RNAs [61,62], which are known to impact nerve regeneration, and should be accordingly considered. In addition, the effect of the exosomal contents on target cells may occur synergistically [63,64]. Hence, an in-depth exploration of mechanisms underlying potential ADSC-exo functions remains urgent.

## 4. Materials and Methods

### 4.1. Cultured Mouse ADSCs

ADSCs were purchased from iXCells Biotechnologies (MADSC-bf, San Diego, CA, USA). The ADSCs were from the interscapular brown fat tissue of C57BL/6 mice. These cells were expanded for subsequent passages using ADSC basal medium (Cat # MD-0003) under the protocol, according to instructions provided by iXcells Biotechnologies. The cells previously tested positive for stem cell markers CD105, CD73, CD90, CD44, and negative for CD3, CD11b, CD25, CD45, and CD106 by flow cytometry analysis. The exosomes secreted by ADSCs (ADSC-exo), as well as ADSCs treated with 100 µg/mL FK506 (InvivoGen, Hong Kong, China) in dimethyl sulfoxide (DMSO) for 24 h (ADSC-F-exo), were isolated for further animal experiments.

### 4.2. Exosome Isolation

Exosomes were purified from the ADSC culture media in the presence or absence of FK506 treatment using the ExoQuick-TC^TM^ exosome precipitation solution (EXOTC50A-1, System Biosciences, Palo Alto, CA, USA), according to the manufacturer’s instructions. Media were centrifuged at 3000× *g* for 15 min, and then the supernatant was transferred into a new tube, followed by the addition of equal volumes of the ExoQuick-TC^TM^ solution. After mixing, supernatants were refrigerated at 4 °C overnight for at least 12 h and then centrifuged at 1500× *g* for 30 min. The supernatant was discarded, the pellet was resuspended in PBS, and used for further experiments.

### 4.3. Characterization of Exosomes

Characterization of isolated exosomes was based on the Guidelines of the Minimal Information for Studies of Extracellular Vesicles (MISEV2018) [65]. Expression of exosomal surface markers on isolated exosomes was detected by western blotting in triplicate, with the culture medium used as control. For exosomes, total protein was separated by polyacrylamide gel electrophoresis and electrotransferred to polyvinylidene fluoride (PVDF) membranes (Millipore, Billerica, MA, USA). The membranes were blocked with 5% skim milk in PBS/Tween-20 and incubated with primary antibodies against CD9 (cat # ab92726, 1:1000; Abcam, Cambridge, MA, USA), CD81 (cat # ab109201, 1:1000; Abcam), Flotillin-1 (cat # 18634, 1:1000; Cell Signaling Technology, Danvers, MA, USA), TSG101 (cat # ab30871, 1:1000; Abcam), and the negative control protein Calnexin (cat # ab22595, 1:1000; Abcam) at 4 °C overnight. Then, membranes were washed with 0.1% TBS/Tween 20 for 10 min, 3 times at room temperature and incubated with horseradish peroxidase (HRP)-conjugated secondary antibodies (cat # NA931; GE Healthcare Amersham, Piscataway, NJ, USA) for 2 h at room temperature; detected proteins were quantified using a FluorChem SP imaging system (Alpha Innotech, San Leandro, CA, USA).

For TEM analyses, 10 µL exosomes were fixed with 2.5% glutaraldehyde for 2 h and added to a 200 mesh Formvar stabilized with carbon. The grids were stained with 2% uranyl acetate for 1 h. Samples were analyzed with a transmission electron microscope HT-7700 (Hitachi, Tokyo, Japan) at 100 kV.

A Zetasizer Nano-ZS DLS system (Malvern, Montréal, QC, Canada) was used to assess the particle diameter of isolated exosomes. In brief, 100 µL of each sample was loaded into an ultraviolet microcuvette (BRAND; Essex, CT, USA) at 4 °C. The Brownian motion of each particle was measured by the fluctuations of scattered light intensity at a wavelength of 633 nm and a fixed angle of 173°. Data points from each replicate represent an average of three automatic measurements of 12–18 runs. The average particle diameter was obtained from the peak of the Gaussian model fit to the particle distribution and presented by PDI [66].

### 4.4. Animal Nerve Crush Surgery

C57BL/6 mice were purchased from the National Laboratory Animal Center, Taiwan. The nerve crush injury model was established in 8–12-week-old male mice, weighing between 20 and 30 g and performed as described in our previous reports [67,68] and by Mackinnon et al. [69,70]. Anesthesia was induced using an intramuscular injection of 25 mg/kg ketamine and 50 mg/kg xylazine. Then, the right sciatic nerve of the mouse was exposed at the mid-thigh level and was crushed with No. 5 Jeweler forceps, using consistent pressure for 30 s. To facilitate the subsequent harvest of the nerve specimen, a 10-0 Ethilon suture (Micro suture Ethicon, Somerville, NJ, USA) was used to mark the injured site only through the epineurium after the release of forceps-induced pressure. There were four groups of nerve samples in this study, including (1), naive nerve; (2), nerve crush control; (3) crush nerve with ADSC-exo treatment; and (4) crush nerve with ADSC-F-exo. For the group 3 and 4, 100 µg ADSC-exo and ADSC-F-exo in 100 µL PBS, respectively, were sprayed around the crushed nerve segment using a 30-gauge syringe needle (Becton-Dickinson & Co, Franklin Lakes, NJ, USA). For the nerve crush control, the crushed nerve segment was sprayed with 100 µL PBS to serve as the treatment control. The left sciatic nerve of those mice in the nerve crush control group was harvested as naïve nerve samples.

The right sciatic nerve of nerve crush control mice was left untouched and used as a control (naïve nerve). Then, all mice were allowed to recover in a separate postoperative care room. At post-crush day 2, mice (*n* = 4) were re-anesthetized to harvest 1 cm of the nerve, distal to the injured site, and were then euthanized. The harvested nerve segment was used for PCR Arrays with subsequent RT-qPCR, as well as for the detection of neurotrophins. At post-crush day 10, additional mice (*n* = 6) were re-anesthetized to harvest 1 cm of the nerve distal to the injured site, followed by euthanasia. The harvested nerve samples were used for histomorphometric analysis and PCR array. Herein, all housing conditions and surgical procedures, analgesia, and assessments were performed in an AAALAC-accredited, specific pathogen-free facility, following national and institutional guidelines. Animal protocols were approved by the IACUC of Chang Gung Memorial Hospital.

### 4.5. Quantitative Assessment of Sciatic Nerve Regeneration

In brief, the axial 1 cm of the nerve distal to the injured site, as well as of the contralateral naïve nerve, for each subgroup (crush nerve + ADSC-exo, crush nerve + ADSC-F-exo, and nerve crush control, *n* = 6 for each subgroup) was isolated and fixed at 4 °C with 3% glutaraldehyde (Polysciences Inc., Warrington, PA, USA), washed in 0.1 M phosphate buffer (pH 7.2), post-fixed with 1% osmium tetroxide (Fisher Scientific, Pittsburgh, PA, USA), dehydrated in graded ethanol solutions, and embedded in Araldite 502 (Polysciences Inc.). Axial semi-thin sections (1-μm-thick nerve specimens), obtained at a 5-mm distance from the injured site, were stained with 1% toluidine blue for histomorphometric analysis. Binary image analysis was performed for semi-automated quantitative analysis of multiple components of nerve histomorphometry in a blinded manner [71]. Total myelinated fiber counts were measured based on six randomly selected fields at 1000 × magnification. The fiber count, fiber width, axon width, fiber area, axon area, myelin area, and total fiber area were calculated.

### 4.6. Gene Expression in RT^2^ Profiler PCR Arrays

Under RNase-Free DNase digestion, total RNA of harvested nerve segments was isolated using the RNeasy Mini kit (Qiagen, Valencia, CA, USA). The RNA integrity was confirmed using an Agilent 2100 BioAnalyzer. A High-Capacity cDNA Reverse Transcription (ABI 4368814, Applied Biosystems, Foster City, CA, USA) was used for reverse transcription. A Mouse Neurogenesis RT^2^ Profiler™ PCR Array (Qiagen) (Table S1), which profiles the expression of 84 genes related to the regulation of key neurogenesis processes such as the cell cycle, cell proliferation, differentiation, motility, and migration, was used to detect the genes expressed in the distal nerve segment on the 7500 Real-Time PCR System (Applied Biosystems, Carlsbad, CA, USA). Quality control was confirmed by the positive PCR controls and reverse transcription controls of the PCR array. The expression level of target genes was calculated according to a panel of housekeeping genes, including beta-actin (*Actb*) and glyceraldehyde-3-phosphate dehydrogenase (*Gapdh*), in the PCR Array. The gene with a threshold cycle above the 34th cycle was excluded from the further comparison. Genes were significantly expressed when a 2-fold differential expression and *p* < 0.05 were detected between experimental and nerve crush control groups.

### 4.7. RT-qPCR

To validate the expression level of differentially expressed genes identified in the PCR array, the converted DNA was subjected to RT-qPCR with specific primers designed for the PCR array by Qiagen using an Applied Biosystems^®^ 7500 Real-Time PCR Systems (Applied Biosystems), with a 96-well optical plate format. Amplification was performed in 25 μL volume reactions containing Power SYBR Green PCR Master Mix (ABI 4367659, Applied Biosystems), according to the manufacturer’s recommendations. The cycling conditions consisted of a 30-min reverse transcription step at 50 °C, a 2 min denaturation step at 95 °C, and 40 amplification cycles of 15 s at 95 °C and 1 min at 60 °C. Fluorescence was acquired during each extension step, and reactions were performed in triplicate. PCR-grade water was used as the negative control. The 2^−ΔΔCt^ formula [72] was used for calculating gene expression, with the endogenous reference gene *Gapdh* used for normalization. Target mRNA levels in nerve crush injuries treated with ADSC-F-exo were measured and compared with those in nerve crush control samples.

### 4.8. Neurotrophin Expression in Nerve Segments

Next, we determined the level of neurotrophins in the nerve segments following exosomal treatment. ELISA was to detect five neurotrophins in nerve segments (crush nerve + ADSC-exo, crush nerve + ADSC-F-exo, and nerve crush control, *n* = 4 for each subgroup) using commercial kits, including NGF (cat# Rab1119, Merck, Darmstadt, Germany), GDNF (cat# ab171178, Abcam, Cambridge, UK), CNTF (cat# CSB-E07312M, Cusabio Technology LLC, Houston, TX, USA), BDNF (cat# KA0331, Abnova, Taipei, Taiwan), and NT-3 (cat# ab213882, Abcam). The neurotrophin concentration was measured according to the calibration curve of the standard sample provided in the kit and presented as pg/mL.

### 4.9. Extraction of Exosomal Protein and iTRAQ Labeling

Exosomal proteins of ADSC-exo and ADSC-F-exo (*n* = 2 for each kind of sample) were purified using the T-PER tissue protein extraction reagent (78510, Thermo Fisher Scientific, Waltham, MA, USA). Protein samples were desalted using the Amicon^®^ Ultra-15 (Merck-Millipore, Burlington, MA, USA) and quantified using the BCA protein assay (23225, Thermo Fisher Scientific). For iTRAQ labeling, 25 µg of the protein samples were dried using SpeedVac and resuspended in the iTRAQ dissolution buffer, which included 0.5 M triethylammonium bicarbonate (TEAB; pH 8.5). Protein samples underwent reduction using the iTRAQ reduction buffer (tris-2-carboxyethyl phosphine, TCEP) at 60 °C for 30 min and were then alkylated in the dark using iodoacetamide at 37 °C for 30 min. After protein digestion using sequencing grade modified trypsin (V511A, Promega, Madison, WI, USA), samples were dried using SpeedVac. Next, the peptides were reconstituted in the iTRAQ dissolution buffer and labeled using iTRAQ labeling reagents, according to the manufacturer’s instructions (Applied Biosystems Inc., Foster City, CA, USA).

### 4.10. Two-Dimensional Liquid Chromatography with Tandem Mass Spectrometry (2D LC-MS/MS)

The iTRAQ-labeled samples were analyzed using the Q Exactive^TM^ HF mass spectrometer (Thermo Fisher Scientific), coupled with the UltiMate™ 3000 RSLCnano HPLC System (Thermo Fisher Scientific). The iTRAQ-labeled peptides were pooled and desalted in Sep-Pak C18 cartridges (Waters, Milford, MA, USA). Then, desalted peptides were dried using SpeedVac and resuspended in 0.5% trifluoroacetic acid. The peptide mixtures were loaded onto an EASY-Spray™ C18 column (Thermo Fisher Scientific)) and separated using 0.1% formic acid solution, with varying amounts of acetonitrile (5~80%). The top 15 abundant precursor ions, within the 375–1400 *m*/*z* scan range, were dynamically selected for further fragmentation in high collision dissociation (HCD) mode, with normalized collision energy set to 33 ± 1. In the full MS scan, the resolution was set to 60,000 at *m*/*z* 200, AGC target to 3e6, and maximum injection time to 50 ms. For the MS/MS scan, the resolution was set to 15,000, AGC target to 5e4, and the maximum injection time to 100 ms. The release of the dynamic exclusion of selected precursor ions was set to 20 s.

### 4.11. Database Search and Protein Quantification

The raw MS data were examined using the Mascot search algorithm (Version 2.5, Matrix Science, Boston, MA, USA) against the Swiss-Prot human protein database with Proteome Discoverer (Version 2.1, Thermo Fisher Scientific) software. For protein identification, the search parameters were set as follows: carbamidomethylation at cysteine as the fixed modification, oxidation at methionine, acetylation at protein N-terminus, iTRAQ-labeled at peptide N-terminus, lysine residue as dynamic modifications, 10 ppm and 0.02 Da for MS/MS tolerance, and a maximum of 2 missing cleavage sites.

### 4.12. Construction of PPI Network and Identification of Hub Proteins

PPI network analysis [73,74] was used to distinguish critical hub proteins among a group of differentially expressed protein targets identified in the iTRAQ experiment. Therefore, the STRING database was used to conduct the PPI network analysis. PPI networks were constructed by Cytoscape 3.6.1, with nodes representing proteins and edges indicating simplifications of interactions between nodes in the network for graphical representation. Using default conditions for the functional enrichment analysis module, the PPI network was used for screening the module based on the MCOD plugin in Cytoscape [75].

### 4.13. Statistical Analysis

All the results were presented as mean ± standard error. An overall analysis of the differences between group means was calculated by one-way analysis of variance (ANOVA), followed by a post-hoc Fisher’s least significant difference test. The statistical significance was set at *p* < 0.05.

## 5. Conclusions

This study revealed that both locally applied ADSC-exo and ADSC-F-exo significantly enhanced nerve regeneration after nerve crush injury. Pretreatment of ADSCs with FK506 failed to produce exosomes carrying more potent molecules for enhanced nerve regeneration. Proteomic analysis of ADSC-exo revealed the notable presence of HDAC, APP, and ITGB1, which may be potential candidates involved in exosome-mediated enhanced nerve regeneration.

## Figures and Tables

**Table 1:** Quantitative histomorphometric analysis of toluidine blue-stained nerve specimens, harvested 5-mm distal to the injured site in C57BL/6 mice on post-crush day 10.

Groups	Fiber Count (*n*)	Fiber Width (μm)	Axon Width (μm)	Fiber Area (μm^2^)	Axon Area (μm^2^)	Myelin Area (μm^2^)	Total Fiber Area (μm^2^)
Naive nerve	110 ± 12	5.40 ± 0.51	3.37 ± 0.40	34.3 ± 4.2	14.3 ± 2.5	17.7 ± 3.2	3592 ± 472
Nerve crush control	86 ± 17	4.08 ± 0.39	2.31 ± 0.31	20.2 ± 6.2	7.2 ± 3.1	11.9 ± 3.1	1782 ± 654
Crush nerve + ADSC-exo	105 ± 19 *	5.02 ± 0.63 *	3.02 ± 0.41 *	28.6 ± 5.4 *	11.3 ± 2.8 *	15.2 ± 3.0 *	2988 ± 528 *
Crush nerve + ADSC-F-exo	108 ± 24 *	4.94 ± 0.77 *	3.09 ± 0.62 *	27.9 ± 7.5 *	11.9 ± 3.2 *	14.5 ± 4.4 *	2822 ± 760 *

Data are shown as mean ± standard error (* indicated *p* < 0.05 when compared to those of crush nerves).

## Data Availability

Not applicable.

## Supplementary Materials

The following are available online at https://www.mdpi.com/article/10.3390/ijms22168545/s1, File S1: List of 192 exosomal proteins noted in the intersection of all four samples in the iTRAQ proteomic analysis. Table S1. Gene table of Mouse Neurogenesis RT^2^ Profiler^TM^ PCR Array.

## References

[1] Armas-Salazar A., García-Jerónimo A.I., Villegas-López F.A., Navarro-Olvera J.L., Carrillo-Ruiz J.D. (2021). Clinical outcomes report in different brachial plexus injury surgeries: A systematic review. Neurosurg. Rev..

[2] BSc M.K.M., Haldane C., Doherty C., Berger M.J. (2021). Evaluation of muscle strength following peripheral nerve surgery: A scoping review. PM&R.

[3] Terzis J.K., Konofaos P. (2013). FK506 and Nerve Regeneration: Past, Present, and Future. J. Reconstr. Microsurg..

[4] Saffari T.M., Bedar M., Zuidam J.M., Shin A.Y., Baan C.C., Hesselink D.A., Hundepool C.A. (2019). Exploring the neuroregenerative potential of tacrolimus. Expert Rev. Clin. Pharmacol..

[5] Zuo K.J., Saffari T.M., Chan K., Shin A.Y., Borschel G.H. (2020). Systemic and Local FK506 (Tacrolimus) and its Application in Peripheral Nerve Surgery. J. Hand Surg..

[6] Bain J.R. (2000). Peripheral nerve and neuromuscular allotransplantation: Current status. Microsurgery.

[7] Mulhall J.P., Klein E.A., Slawin K., Henning A.K., Scardino P.T. (2018). A Randomized, Double-Blind, Placebo-Controlled Trial to Assess the Utility of Tacrolimus (FK506) for the Prevention of Erectile Dysfunction Following Bilateral Nerve-Sparing Radical Prostatectomy. J. Sex. Med..

[8] Glaus S.W., Johnson P.J., Mackinnon S.E. (2011). Clinical Strategies to Enhance Nerve Regeneration in Composite Tissue Allotransplantation. Hand Clin..

[9] Yan Y., Sun H.H., Hunter D.A., MacKinnon S.E., Johnson P.J. (2012). Efficacy of Short-Term FK506 Administration on Accelerating Nerve Regeneration. Neurorehabilit. Neural Repair.

[10] Kubiak C.A., Grochmal J., Kung T.A., Cederna P.S., Midha R., Kemp S.W. (2019). Stem-cell–based therapies to enhance peripheral nerve regeneration. Muscle Nerve.

[11] Shin A.Y., Saffari S., Saffari T.M., Ulrich D.J.O., Hovius S.E.R. (2021). The interaction of stem cells and vascularity in peripheral nerve regeneration. Neural Regen. Res..

[12] Sayad-Fathi S., Nasiri E., Zaminy A. (2019). Advances in stem cell treatment for sciatic nerve injury. Expert Opin. Biol. Ther..

[13] Chan T.-M., Chen J.Y.-R., Ho L.-I., Lin H.-P., Hsueh K.-W., Liu D.D., Chen Y.-H., Hsieh A.-C., Tsai N.-M., Hueng D.-Y. (2014). ADSC Therapy in Neurodegenerative Disorders. Cell Transplant..

[14] Rosén A., Tardast A., Shi T.-J. (2016). How Far Have We Come in the Field of Nerve Regeneration After Trigeminal Nerve Injury?. Curr. Oral Health Rep..

[15] Selaru A., Dinescu S., Costache M. (2020). The Cellular and Molecular Patterns Involved in the Neural Differentiation of Adipose-Derived Stem Cells. Cell Biol. Transl. Med..

[16] Yan H., Ding Y., Lu M. (2020). Current Status and Prospects in the Treatment of Erectile Dysfunction by Adipose-Derived Stem Cells in the Diabetic Animal Model. Sex. Med. Rev..

[17] Kingham P.J., Kolar M.K., Novikova L.N., Novikov L., Wiberg M. (2014). Stimulating the Neurotrophic and Angiogenic Properties of Human Adipose-Derived Stem Cells Enhances Nerve Repair. Stem Cells Dev..

[18] Sowa Y., Imura T., Numajiri T., Nishino K., Fushiki S. (2012). Adipose-Derived Stem Cells Produce Factors Enhancing Peripheral Nerve Regeneration: Influence of Age and Anatomic Site of Origin. Stem Cells Dev..

[19] Greening D.W., Xu R., Ji H., Tauro B.J., Simpson R.J. (2015). A Protocol for Exosome Isolation and Characterization: Evaluation of Ultracentrifugation, Density-Gradient Separation, and Immunoaffinity Capture Methods. Methods Mol. Biol..

[20] Lin J., Liu J., Huang B., Chen X., Chen X.-M., Xu Y.-M., Huang L.-F., Wang X.-Z. (2015). Exosomes: Novel Biomarkers for Clinical Diagnosis. Sci. World J..

[21] Farinazzo A., Turano E., Marconi S., Bistaffa E., Bazzoli E., Bonetti B. (2015). Murine adipose-derived mesenchymal stromal cell vesicles: In vitro clues for neuroprotective and neuroregenerative approaches. Cytotherapy.

[22] Bucan V., Vaslaitis D., Peck C.-T., Strauß S., Vogt P.M., Radtke C. (2018). Effect of Exosomes from Rat Adipose-Derived Mesenchymal Stem Cells on Neurite Outgrowth and Sciatic Nerve Regeneration After Crush Injury. Mol. Neurobiol..

[23] Huang X., Ding J., Li Y., Liu W., Ji J., Wang H., Wang X. (2018). Exosomes derived from PEDF modified adipose-derived mesenchymal stem cells ameliorate cerebral ischemia-reperfusion injury by regulation of autophagy and apoptosis. Exp. Cell Res..

[24] Chen J., Laramore C., Shifman M.I. (2016). Differential expression of HDACs and KATs in high and low regeneration capacity neurons during spinal cord regeneration. Exp. Neurol..

[25] Cho Y., Cavalli V. (2014). HDAC signaling in neuronal development and axon regeneration. Curr. Opin. Neurobiol..

[26] Dzreyan V.A., Rodkin S.V., Pitinova M.A., Uzdensky A.B. (2020). HDAC1 Expression, Histone Deacetylation, and Protective Role of Sodium Valproate in the Rat Dorsal Root Ganglia after Sciatic Nerve Transection. Mol. Neurobiol..

[27] Ganai S.A., Ramadoss M., Mahadevan V. (2016). Histone Deacetylase (HDAC) Inhibitors—Emerging roles in neuronal memory, learning, synaptic plasticity and neural regeneration. Curr. Neuropharmacol..

[28] Palmisano I., Di Giovanni S. (2018). Advances and Limitations of Current Epigenetic Studies Investigating Mammalian Axonal Regeneration. Neurotherapeutics.

[29] Tang B.L. (2014). Class II HDACs and Neuronal Regeneration. J. Cell. Biochem..

[30] Wang M.-H., Wu C.-H., Huang T.-Y., Sung H.-W., Chiou L.-L., Lin S.-P., Lee H.-S. (2019). Nerve-mediated expression of histone deacetylases regulates limb regeneration in axolotls. Dev. Biol..

[31] Wu L.M.N., Wang J., Conidi A., Zhao C., Wang H., Ford Z., Zhang L., Zweier C., Ayee B.G., Maurel B.G.A.P. (2016). Zeb2 recruits HDAC–NuRD to inhibit Notch and controls Schwann cell differentiation and remyelination. Nat. Neurosci..

[32] Li W., Tam K.M.V., Chan W.W., Koon A.C., Ngo J.C.K., Chan H.Y.E., Lau K.-F. (2018). Neuronal adaptor FE65 stimulates Rac1-mediated neurite outgrowth by recruiting and activating ELMO1. J. Biol. Chem..

[33] Soininen H.S., Riekkinen P.J. (1996). Apolipoprotein E, memory and Alzheimer’s disease. Trends Neurosci..

[34] Yun H.-M., Park K.-R., Kim E.-C., Kim S., Hong J.T. (2015). Serotonin 6 receptor controls alzheimer’s disease and depression. Oncotarget.

[35] Cohen J., Johnson A.R. (1991). Differential effects of laminin and merosin on neurite outgrowth by developing retinal ganglion cells. J. Cell Sci..

[36] Kuo Y.-C., Shih-Huang C.-Y., Rajesh R. (2021). Enhanced integrin affinity and neural differentiation of induced pluripotent stem cells using Ln5-P4-grafted amphiphilic solid lipid nanoparticles. Mater. Sci. Eng. C.

[37] Mathews G.A., Ffrench-Constant C. (1995). Embryonic fibronectins are up-regulated following peripheral nerve injury in rats. J. Neurobiol..

[38] Piraino P., Yednock T., Messersmith E., Pleiss M., Freedman S., Hammond R., Karlik S. (2005). Spontaneous remyelination following prolonged inhibition of α4 integrin in chronic EAE. J. Neuroimmunol..

[39] Sakaguchi D., Radke K. (1996). β1 integrins regulate axon outgrowth and glial cell spreading on a glial-derived extracellular matrix during development and regeneration. Dev. Brain Res..

[40] Taskinen H.-S., Heino J., Röyttä M. (1995). The dynamics of β1 integrin expression during peripheral nerve regeneration. Acta Neuropathol..

[41] Kuo M.H., Allis C.D. (1998). Roles of histone acetyltransferases and deacetylases in gene regulation. Bioessays.

[42] Hubbert C., Guardiola A., Shao R., Kawaguchi Y., Ito A., Nixon A., Yoshida M., Wang X.-F., Yao T.-P. (2002). HDAC6 is a microtubule-associated deacetylase. Nat. Cell Biol..

[43] Tea J., Chihara T., Luo L. (2010). Histone Deacetylase Rpd3 Regulates Olfactory Projection Neuron Dendrite Targeting via the Transcription Factor Prospero. J. Neurosci..

[44] Montgomery R.L., Hsieh J., Barbosa A.C., Richardson J.A., Olson E.N. (2009). Histone deacetylases 1 and 2 control the progression of neural precursors to neurons during brain development. Proc. Natl. Acad. Sci. USA.

[45] Nott A., Watson P.M., Robinson J.D., Crepaldi L., Riccio A. (2008). S-nitrosylation of histone deacetylase 2 induces chromatin remodelling in neurons. Nat. Cell Biol..

[46] Chen Y., Wang H., Yoon S.O., Xu X., Hottiger M., Svaren J., Nave K.A., Kim H.A., Olson E.N., Lu Q.R. (2011). HDAC-mediated deacetylation of NF-κB is critical for Schwann cell myelination. Nat. Neurosci..

[47] Weingarten J., Weingarten M., Wegner M., Volknandt W. (2017). APP—A Novel Player within the Presynaptic Active Zone Proteome. Front. Mol. Neurosci..

[48] Li H.L., Roch J.M., Sundsmo M., Otero D., Sisodia S., Thomas R., Saitoh T. (1997). Defective neurite extension is caused by a mutation in amyloid beta/A4 (A beta) protein precursor found in familial Alzheimer’s disease. J. Neurobiol..

[49] Jin L.W., Ninomiya H., Roch J.M., Schubert D., Masliah E., Otero D.A., Saitoh T. (1994). Peptides containing the RERMS sequence of amyloid beta/A4 protein precursor bind cell surface and promote neurite extension. J. Neurosci..

[50] Kotulska K., Larysz-Brysz M., LePecheur M., Marcol W., Lewin-Kowalik J., Paly E., London J. (2010). APP overexpression prevents neuropathic pain and motoneuron death after peripheral nerve injury in mice. Brain Res. Bull..

[51] Kotulska K., Larysz-Brysz M., LePecheur M., Marcol W., Olakowska E., Lewin-Kowalik J., London J. (2011). APP/SOD1 overexpressing mice present reduced neuropathic pain sensitivity. Brain Res. Bull..

[52] Hu X., Hu J., Dai L., Trapp B., Yan R. (2015). Axonal and Schwann Cell BACE1 Is Equally Required for Remyelination of Peripheral Nerves. J. Neurosci..

[53] Farah M.H. (2012). BACE1 influences debris clearance and axonal regeneration in injured peripheral nerve. J. Peripher. Nerv. Syst..

[54] Ruoslahti E. (1991). Integrins. J. Clin. Investig..

[55] Yao L., Damodaran G., Nikolskaya N., Gorman A., Windebank A., Pandit A. (2009). The effect of laminin peptide gradient in enzymatically cross-linked collagen scaffolds on neurite growth. J. Biomed. Mater. Res. Part A.

[56] Siironen J., Sandberg M., Vuorinen V., Roytta M. (2006). Laminin B1 and Collagen Type IV Gene Expression in Transected Peripheral Nerve: Reinnervation Compared to Denervation. J. Neurochem..

[57] Skup M. (2018). Neurotrophins: Evolution of concepts on rational therapeutic approaches. Postepy Biochem..

[58] Gordon T. (2009). The role of neurotrophic factors in nerve regeneration. Neurosurg. Focus.

[59] Tang X., Sun C. (2020). The roles of MicroRNAs in neural regenerative medicine. Exp. Neurol..

[60] Ren Z.-W., Zhou J.-G., Xiong Z.-K., Zhu F.-Z., Guo X.-D. (2019). Effect of exosomes derived from MiR-133b-modified ADSCs on the recovery of neurological function after SCI. Eur. Rev. Med. Pharmacol. Sci..

[61] He L., Zhu C., Jia J., Hao X.-Y., Yu X.-Y., Liu X.-Y., Shu M.-G. (2020). ADSC-Exos containing MALAT1 promotes wound healing by targeting miR-124 through activating Wnt/β-catenin pathway. Biosci. Rep..

[62] Patel N.A., Moss L.D., Lee J.-Y., Tajiri N., Acosta S., Hudson C., Parag S., Cooper D.R., Borlongan C.V., Bickford P.C. (2018). Long noncoding RNA MALAT1 in exosomes drives regenerative function and modulates inflammation-linked networks following traumatic brain injury. J. Neuroinflam..

[63] Hoshino D., Kirkbride K.C., Costello K., Clark E.S., Sinha S., Grega-Larson N., Tyska M.J., Weaver A.M. (2013). Exosome Secretion Is Enhanced by Invadopodia and Drives Invasive Behavior. Cell Rep..

[64] Salimi L., Akbari A., Jabbari N., Mojarad B., Vahhabi A., Szafert S., Kalashani S.A., Soraya H., Nawaz M., Rezaie J. (2020). Synergies in exosomes and autophagy pathways for cellular homeostasis and metastasis of tumor cells. Cell Biosci..

[65] Théry C., Witwer K.W., Aikawa E., Alcaraz M.J., Anderson J.D., Andriantsitohaina R., Antoniou A., Arab T., Archer F., Atkin-Smith G.K. (2018). Minimal information for studies of extracellular vesicles 2018 (MISEV2018): A position statement of the International Society for Extracellular Vesicles and update of the MISEV2014 guidelines. J. Extracell. Vesicles.

[66] Maroto R., Zhao Y., Jamaluddin M., Popov V.L., Wang H., Kalubowilage M., Zhang Y., Luisi J., Sun H., Culbertson C.T. (2017). Effects of storage temperature on airway exosome integrity for diagnostic and functional analyses. J. Extracell. Vesicles.

[67] Hsieh C.-H., Rau C.-S., Kuo P.-J., Liu S.-H., Wu C.-J., Lu T.-H., Wu Y.-C., Lin C.-W. (2017). Knockout of toll-like receptor impairs nerve regeneration after a crush injury. Oncotarget.

[68] Wu S.-C., Rau C.-S., Lu T.-H., Wu C.-J., Wu Y.-C., Tzeng S.-L., Chen Y.-C., Hsieh C.-H. (2013). Knockout of TLR4 and TLR2 impair the nerve regeneration by delayed demyelination but not remyelination. J. Biomed. Sci..

[69] Magill C.K., Tong A., Kawamura D., Hayashi A., Hunter D.A., Parsadanian A., MacKinnon S.E., Myckatyn T.M. (2007). Reinnervation of the tibialis anterior following sciatic nerve crush injury: A confocal microscopic study in transgenic mice. Exp. Neurol..

[70] Genden E.M., Watanabe O., Mackinnon S.E., Hunter D.A., Strasberg S.R. (2002). Peripheral Nerve Regeneration in the Apolipoprotein-E-Deficient Mouse. J. Reconstr. Microsurg..

[71] Hunter D.A., Moradzadeh A., Whitlock E., Brenner M.J., Myckatyn T.M., Wei C.H., Tung T.H., Mackinnon S.E. (2007). Binary imaging analysis for comprehensive quantitative histomorphometry of peripheral nerve. J. Neurosci. Methods.

[72] Dundas J., Ling M. (2012). Reference genes for measuring mRNA expression. Theory Biosci..

[73] Athanasios A., Charalampos V., Vasileios T., Ashraf G.M. (2017). Protein-Protein Interaction (PPI) Network: Recent Advances in Drug Discovery. Curr. Drug Metab..

[74] Murakami Y., Tripathi L.P., Prathipati P., Mizuguchi K. (2017). Network analysis and in silico prediction of protein–protein interactions with applications in drug discovery. Curr. Opin. Struct. Biol..

[75] Bader G.D., Hogue C.W.V. (2003). An automated method for finding molecular complexes in large protein interaction networks. BMC Bioinform..

